# How Do Novel M-Rootstock (*Vitis* Spp.) Genotypes Cope with Drought?

**DOI:** 10.3390/plants9101385

**Published:** 2020-10-17

**Authors:** Davide Bianchi, Leila Caramanico, Daniele Grossi, Lucio Brancadoro, Gabriella De Lorenzis

**Affiliations:** Dipartimento di Scienze Agrarie ed Ambientali, Università degli Studi di Milano, via Giovanni Celoria 2, 20133 Milano, Italy; davide.bianchi3@unimi.it (D.B.); leila.caramanico@unimi.it (L.C.); grossi.lele@gmail.com (D.G.)

**Keywords:** ABA biosynthesis, ABA signaling, photosynthetic activity, stem water potential, stomatal conductance, transpiration, water use efficiency

## Abstract

Most of the vineyards around the world are in areas characterized by seasonal drought, where water deficits and high temperatures represent severe constraints on the regular grapevine growth cycle. Although grapevines are well adapted to arid and semi-arid environments, water stress can cause physiological changes, from mild to irreversible. Screening of available *Vitis* spp. genetic diversity for new rootstock breeding programs has been proposed as a way for which new viticulture challenges may be faced. In 2014, novel genotypes (M-rootstocks) were released from the University of Milan. In this work, the behavior of M1, M3 and M4 in response to decreasing water availabilities (80%, 50% and 20% soil water content, SWC) was investigated at the physiological and gene expression levels, evaluating gas exchange, stem water potential and transcript abundances of key genes related to ABA (abscisic acid) biosynthesis (*VvZEP*, *VvNCED1* and *VvNCED2*) and signaling (*VvPP2C4*, *VvSnRK2.6* and *VvABF2*), and comparing them to those of cuttings of nine commercial rootstocks widely used in viticulture. M-rootstocks showed a change at physiological levels in severe water-stressed conditions (20% soil water content, SWC), reducing the stomatal conductance and stem water potential, but maintaining high photosynthetic activity. Water use efficiency was high in water-limiting conditions. The transcriptional changes were observed at 50% SWC, with an increment of transcripts of *VvNCED1* and *VvNCED2* genes. M-rootstocks showed similar behavior to 1103P and 110R rootstocks, two highly tolerant commercial genotypes. These rootstocks adopted a *tolerant* strategy to face water-stressed conditions.

## 1. Introduction

Grapevine (*Vitis vinifera* L.) is one of the most widely cultivated and prized fruit crops around the world. In arid and semi-arid environments, the vines undergo a slow decrease in water availability during the growing season [1]. Traditionally, grapevine is a non-irrigated crop due to the adaptation to water limited conditions, though severe water stress causes minor to irreversible physiological and biochemical changes [2,3].

World viticulture is characterized by the use of *V. vinifera* varieties grafted onto a rootstock (*Vitis* spp.) due to the arrival of phylloxera (*Daktulosphaira vitifoliae* (Fitch)), a severe threat for grapevine survival, which was accidentally imported into Europe from North America [4]. North American *Vitis* species are able to resist to phylloxera due to having co-evolved with the pathogen, therefore they are utilized as rootstocks, as single or inter-specific hybrids. Rootstocks also contribute to the control of other soil-borne pests such as nematodes, as well as various abiotic constraints, such as drought, salinity, lime-rich soils and deficient mineral nutrition [5,6,7,8]. They also modify whole plant development, biomass accumulation and phenology [9]. 

The Mediterranean basin is considered one of the most vulnerable regions of the world to climate change and will potentially have to deal with water scarcity and soil erosion in the next few years [10,11]. Its climate is characterized by infrequent rainfall (less than 100 days per year) that is unevenly distributed over time (long periods of summer drought) and sometimes quite sparse (about 300 to 500 mm per year in some semi-arid regions). Most climate change scenarios for this area predict a decrease in rainfall and higher temperatures. IPCC (Intergovernmental Panel on Climate Change) forecasts indicate a yearly temperature increase between 2 °C and 4 °C and a decrease in rainfall between 4% and 30% by 2050 [12]. Due to their perennial status, grapevines will be highly vulnerable to environmental changes, representing a substantial risk for viticulture [13]. 

Water flows into the plant in a soil–plant–atmosphere continuum [14]. The whole water transport system in the plant is influenced by the anatomical structure of xylem vessels [15], hydraulic constraints [16] and chemical signals [17,18]. When soil water availability decreases, one of the earliest responses is stomatal closure, in order to maintain a favorable water balance, buffering the drop in xylem water potential and avoiding embolisms [19,20]. The closure of guard cells leads to a reduction of CO_2_ assimilation and H_2_O transpiration from leaves and, consequently, the photosynthetic activity decreases sharply [21].

One of the factors inducing stomatal closure is abscisic acid (ABA), a hormone produced by roots and leaves [22,23,24,25,26,27,28,29,30]. ABA accumulates in the plant when soil dries out and water potential drops [22], the synthesis of which is entrusted to a minor branch of the carotenoid pathway. The early steps of ABA biosynthesis are catalyzed by zeaxanthin epoxidase (ZEP) and 9-*cis*-epoxycarotenoid dioxygenase (NCED) enzymes [31]. *VvZEP* and *VvNCED* gene expression is strongly induced by water stress [32,33,34] and salt stress [35]. This hormone, through the xylem sap, reaches guard cells, enhancing the content of reactive oxygen species (ROS, especially H_2_O_2_). Stopping the influx and promoting the efflux of potassium ions (K+) results in a rise in calcium ions (Ca^2+^) in the cytosol and, consequently, cells lose their turgor. The ABA signaling pathway is mediated by three main components: (i) pyrabactin resistance1/pyr1-like/regulatory components of ABA receptors (PYR/PYL/RCAR family of ABA receptors); (ii) ABA-regulated protein phosphatase 2Cs (PP2CAs); (iii) ABA-regulated SNRK2 protein kinase (SnPK2) [36,37]. Without stimuli, the ABA receptor is an unliganded form and the protein kinase is bound to the protein phosphatase. Specific receptors (PYR/PYL/RCARs) bind to ABA when its concentration increases and the hormone–receptor complex becomes an active site for the protein PP2C. The activated receptor binds to PP2C and frees SnPK2, which in turn is phosphorylated by another protein kinase. Multiple step phosphorylation of SnRK2 activates ABRE-binding protein (ABRB)/ABRE-binding factor (ABF) which induces many ABA-responsive gene expression [38]. 

In grapevine, the expression of *VvNCED1*, *VvNCED2* and *VvZEP* genes has been directly correlated with ABA accumulation in response to water stress [33,34,39] and their expression was suggested as marker of ABA biosynthesis [40]. The expression of genes involved in the ABA signaling pathway revealed that the genes coding for RCAR, SnRK and ABF are downregulated in drought conditions, while *VvPP2C* genes are generally upregulated [40,41].

In the context of global warming, the exploitation of grapevine genetic diversity and the better understanding of plant responses to environmental stresses represent the way in which new viticultural challenges may be faced [42,43,44]. Although a significant number of efforts in grapevine rootstock selection have been carried out to date, fewer than 10 rootstocks are widely used in viticulture, with a negative impact on the grapevine response to biotic and abiotic stresses [9,45]. Since 1985, the Department of Agricultural and Environmental Sciences (DiSAA) research group operating at the University of Milan has been working on the selection of new rootstocks able to cope with abiotic stresses [5]. Some genotypes (series “M”: M1, M2, M3 and M4) were selected and released in 2014 and registered in the National Register of Vine Varieties (RNVV). M1 and M3 exhibit tolerance to iron-limited conditions (M1 > M3) [8,46], M2 and M4 display moderate resistance to salinity (Porro et al., 2013; Meggio et al., 2014) and M4 shows high tolerance to drought (Porro et al., 2013; Meggio et al., 2014; Corso et al., 2015). 

To assess the drought tolerance of M-rootstocks in comparison to other commercial genotypes largely used in viticulture, physiological (gas exchange and stem water potential) and transcriptomic performances (genes involved in ABA synthesis and ABA-mediated responses to drought) were evaluated under well-watered and water-stressed conditions. 

## 2. Results

### 2.1. The Physiological Response of Grapevine Rootstocks to Drought

The physiological parameters of photosynthesis (Pn), stomatal conductance (Gs), transpiration (E) and stem water potential (Ψ_S_) were evaluated in 12 own-rooted grapevine rootstocks under decreasing water availability (from 80 to 20% soil water content, SWC) (Table 1, Table S1). 

**Figure 1:**
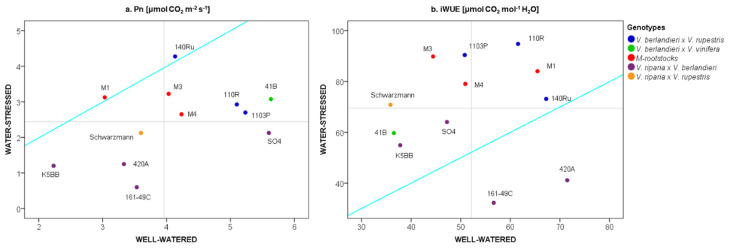
Comparison of performances in 12 own-rooted grapevine rootstocks under both well-watered (80% soil water content, SWC) and water-stressed (20% SWC) conditions in terms of net photosynthesis (Pn) (**a**) and intrinsic water use efficiency (iWUE) (**b**). Colors are attributed according to the breeding materials (Table 1). M-rootstock pedigree: M1—K5BB × Teleki 5C; M3—K5BB × Teleki 5C; M4—unknown × 1103P. Lines are set to the mean values of Pn (**a**) and iWUE (**b**) for each water condition. Lines 1:1 are reported in cyan.

The physiological activity reported in well-watered conditions (80% SWC) was maintained at 50% SWC and decreased at 20% SWC (Figure S1). The water condition showing the most significant differences (20% SWC) was used to investigate the behavior of each grapevine rootstock under water deficit conditions, in terms of photosynthetic activity and intrinsic water use efficiency (iWUE) (Figure 1). The *V. berlandieri × V. rupestris* hybrids (1103P, 110R and 140Ru rootstocks), 41B, M4 and M3 rootstocks carried out high photosynthetic activity under both water conditions, exceeding average levels. The *V. riparia* × *V. berlandieri* hybrids (161-49C, 420A, K5BB) showed Pn values lower than average values at both water availabilities. The M1 rootstock showed similar Pn values in both conditions, exceeding the average value at 20% SWC (Figure 1a). Differences between genotypes also occurred when iWUE, calculated as the ratio between Pn and stomatal conductance values, was taken into account: 110R, 140Ru and M1 rootstocks maintained high efficiency when SWC decreased; iWUE values of 161-49C were reduced at 20% SWC; the 41B, K5BB and SO4 rootstocks reported iWUE values under average levels at 80% SWC, maintaining the same efficiency at 20%; 1103P, M3 and M4 rootstocks increased their efficiency under the water-stressed condition (Figure 1b).

**Figure 2:**
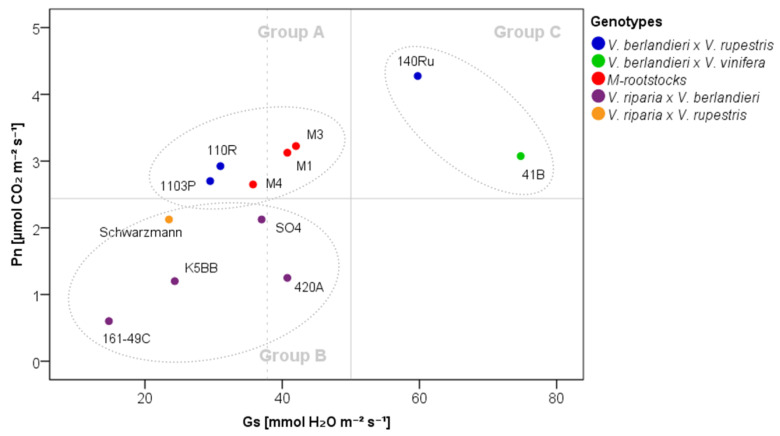
Stomatal conductance (Gs) as function of net photosynthesis (Pn) in 12 own-rooted grapevine rootstocks at and 20% soil water content (SWC). Colors are attributed according to the breeding materials (Table 1). M-rootstock pedigree: M1—K5BB × Teleki 5C; M3—K5BB × Teleki 5C; M4—unknown × 1103P. Thresholds for group identification were set to 50 mmol H_2_O m^−2^ s^−1^ [47] for Gs and to the average for Pn at 20% SWC. The dotted line shows the average Gs value at 20% SWC.

To investigate rootstock WUE in depth, Pn was analyzed as a function of Gs under the water-stressed condition (20% SWC). Clear differences emerged in the behavior of the 12 genotypes, resulting in three different groups (Figure 2): (i) Group A, genotypes reporting Gs values lower than the water-stressed threshold (50 mmol H_2_O m^−2^ s^−1^, based on Cifre et al. [47]) and Pn values higher than the general average value (2.5 µmol CO_2_ m^−2^ s^−1^) (1103P, 110R, M1, M3 and M4 rootstocks); (ii) Group B, genotypes reporting Gs values lower than the water-stressed threshold and Pn values lower than the general average value (161-49C, 420A, K5BB, Schwarzmann and SO4 rootstocks); (iii) Group C, genotypes reporting Gs values higher than the water-stressed threshold and Pn values higher than the general average value (140Ru and 41B rootstocks). 

**Figure 3:**
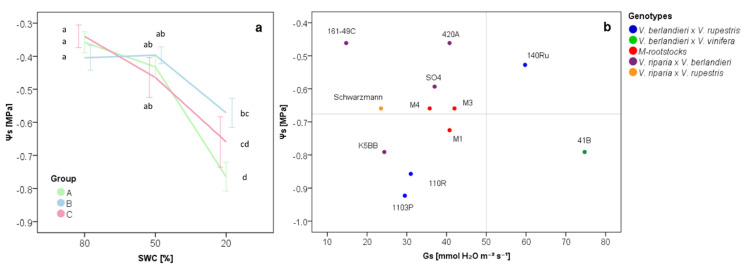
Stem water potential (Ψ_S_) as a function of decreasing soil water content (SWC) (**a**) and stomatal conductance (**b**). Groups are defined in Figure 2, according to the gas exchange values. Group A: 1103P, 110R, M1, M3 and M4 rootstocks; Group B: 161-49C, 420A, K5BB, Schwarzmann and SO4 rootstocks; Group C: 140Ru and 41B rootstocks. Letters show the statistical differences defined according to Tukey’s post hoc test at a *p*-value of 0.05. In plot (b), thresholds were set to 50 mmol H_2_O m^−2^ s^−1^ [47] for Gs and to the average for Ψ_S_ at 20% SWC.

The three rootstock groups (A, B and C) were compared in term of Ψ_S_ with the decreasing levels of SWC (80%, 50% and 20%). Stem water potential settled at −0.4 MPa at 80% and 50% SWC without showing statistically significant differences among groups. At 20% SWC, Group A and C rootstocks decreased in Ψ_S_ value, whereas Group B rootstocks maintained higher Ψ_S_ values, without a significant reduction of Ψ_S_ values with respect to 50% SWC. At 20% SWC, Group A rootstocks reported Ψ_S_ values lower than Group B rootstocks (Figure 3a). Moreover, the Ψ_S_ was analyzed as a function of stomatal conductance and differences among groups were identified as well (Figure 3b): Group A rootstocks showed, mainly, Gs and Ψ_S_ levels below the stress threshold (50 mmol H_2_O m^−2^ s^−1^, based on Cifre et al. [47]) and average value, respectively; Group B rootstocks showed Gs values below the threshold and, mainly, Ψ_S_ values above the average value (except for K5BB rootstocks); Group C rootstocks showed Gs values exceeding the stress threshold, whereas the Ψ_S_ value was higher than the average for 140Ru rootstock and lower than the average for 41B rootstock. 

At 20% SWC, groups were compared for all physiological parameters and the results are summarized in Table 2. Group B rootstocks significantly differed from Group A and C rootstocks for Pn and Ψ_S_ values, while Group C rootstocks significantly differed from Group A and B rootstocks for Gs and E values.

### 2.2. The Transcriptional Response of Grapevine Rootstocks to Drought

**Figure 4:**
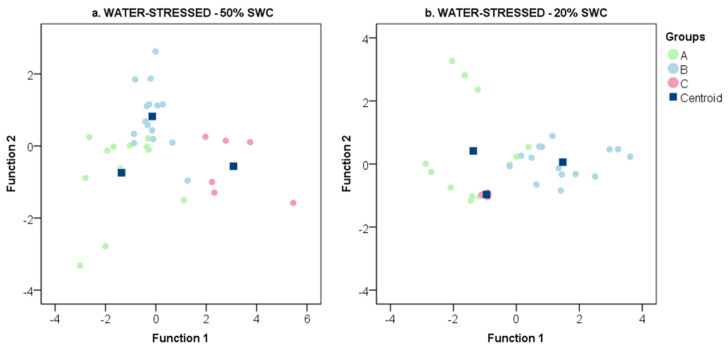
Discriminant analysis of transcript (*VvZEP, VvNCED1, VvNCED2, VvPP2C4, VvSnRK2.6* and *VvABF2* genes) abundance data detected for 12 own-rooted grapevine rootstocks grown under limited water conditions. (**a**) Data collected at 50% soil water content (SWC). (**b**) Data collected at 20% SWC. The genotypes are classified into three groups (A, B and C), as defined in Figure 2, according to the intrinsic water use efficiency. Group A: 1103P, 110R, M1, M3 and M4 rootstocks; Group B: 161-49C, 420A, K5BB, Schwarzmann and SO4 rootstocks; Group C: 140Ru and 41B rootstocks. Discriminant function coefficients are reported in Table S3.

Based on the physiological behavior presented in Figure 2, the gene expression values (*VvNCED1*, *VvNCED2*, *VvZEP* in roots and *VvPP2C4, VvSnRK2.6, VvABF2 in* leaves) were compared among the three groups (A, B and C) by discriminant analysis (Figure 4). Average values and standard error of gene expression for six genes analyzed are reported in Table S2. At 50% SWC, the three groups showed a different transcriptional behavior: Group A and C rootstocks were discriminated along the first function (*p* = 0.000), while Group A and B rootstocks were discriminated along both the first (*p* = 0.034) and the second (*p* = 0.000) functions (Figure 4a). Functions 1 and 2 accounted for 81.0% and 19.0% of total variability, respectively. The most discriminating variables among the groups were the *VvABF2* gene for function 1 and *VvNCED1* and *VvNCED2* genes for function 2. Function 1 was significantly correlated to *VvABF2* (0.350) and *VvNCED2* (−0.105) gene expression values and function 2 to *VvZEP* (−0.346), *VvNCED1* (−0.644), *VvSnRK2.6* (0.443) and *VvPP2C4* (0.314) gene expression values. At 20% SWC, Group A and C rootstocks showed a similar behavior for the first function (0.881), different from the one shown by Group B rootstocks. Group B rootstocks were discriminated along the first function from Group A (*p* = 0.000) and C (*p* = 0.000) rootstocks (Figure 4b). The second function discriminated Groups A and C (0.021). Functions 1 and 2 accounted for 88.6% and 11.4% of total variability, respectively. The most discriminating variables among groups were *VvNCED1* and *VvNCED2* genes for function 1 and *VvSnRK2.6* and *VvZEP* genes for function 2. Function 1 reported significant and positive correlations to *VvPP2C4* (0.394) and *VvABF* (0.234) gene expression values, whereas function 2 reported significant and positive correlations to *VvZEP* (0.801), *VvNCED1* (0.872), *VvNCED2* (0.499) and *VvSnRK2.6* (0.156) gene expression values. 

**Figure 5:**
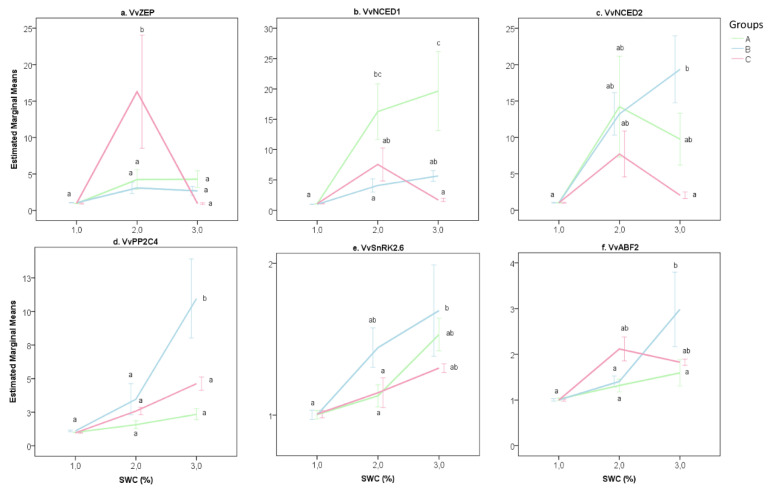
Graphical representation of gene expression data of six genes related to the ABA metabolism in roots (**a**–**c**: *VvZEP, VvNCED1* and *VvNCED2*) and leaves (**d**–**f**: *VvPP2C4, VvSnRK2.6* and *VvABF2*) of 12 own-rooted grapevine rootstocks grown under limited water conditions (from 80 to 20% of soil water content, SWC). The genotypes are classified into three groups (A, B and C), as defined in Figure 2, according to the intrinsic water use efficiency. Average values and standard error are shown. Statistical differences are defined according to Tukey’s post hoc test at a *p*-value of 0.05. Group A: 1103P, 110R, M1, M3 and M4; Group B: 161-49C, 420A, K5BB, Schwarzmann and SO4; Group C: 140Ru and 41B.

In Figure 5, the gene expression trend of each gene for each group is shown. The *VvZEP* gene showed a significant increment of transcripts only for Group C rootstocks (Figure 5a). For the *VvNCED1* gene, the gene expression levels increased significantly at 50% SWC and reached the highest value at 20% SWC for Group A rootstocks (Figure 5b). For *VvPP2C4*, *VvSnK2.6* and *VvABF2* genes, Group B rootstocks showed a significant increment of transcripts at 20% SWC (Figure 5d–f). 

**Figure 6:**
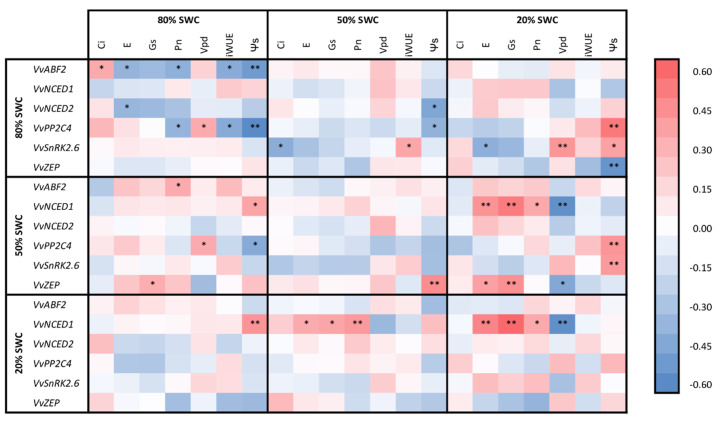
Heatmap of correlation matrix (Pearson index) among physiological (E, Gs, Pn and Ψ_S_) and transcriptional (*VvZEP, VvNCED1, VvNCED2, VvPP2C4, VvSnRK2.6* and *VvABF2* genes) data detected for 12 own-rooted grapevine rootstocks grown under limited water conditions (from 80 to 20% soil water content). E: Transpiration; Gs: Stomatal conductance; Pn: Photosynthetic activity; Ψ_S_: Stem water potential. Statistically significant differences are reported at 0.05 (*) and 0.01 (**) levels.

In Figure 6, gas exchange (Pn, Gs, E) detected at 20% SWC showed a significant negative correlation to the expression of the gene *VvNCED1* obtained at 20% and 50% SWC. Transpiration and stomatal conductance also showed a negative correlation to *VvZEP* at 50% SWC. Vpd at 20% SWC correlated to *VvNCED1* and *VvZEP* expressed at 50% SWC and to *VvNCED1* expressed at 20% SWC. Ψs detected at 20% SWC showed a positive correlation to *VvZEP* at 80% SWC, but a negative correlation to *VvPP2C4* and *VvSnRK2.6* at both 80% and 50% SWC. 

## 3. Discussion 

### 3.1. Water-Limiting Conditions for Grapevine Rootstocks

Grapevines can easily face conditions of mild water stress without their physiological activity being affected, allowing these plants to grow in many marginal areas, usually characterized by limited soil water availability. Roots are the major interface between plants and soil and the first organ to perceive water availability. They are involved in activating key steps for triggering a drought reaction to water stress: signal perception, signal transduction to shoots and leaves and water stress-inducible gene expression (Lovisolo et al., 2016). Therefore, rootstocks play an essential role in the water stress response in grapevines.

In this study, the short-term response to drought of three new-generation (M1, M3 and M4) and nine commercial rootstocks was evaluated. At the physiological level, soil water capacity at 50% was not a limiting condition for M-rootstocks and the nine commercial rootstocks analyzed, with no statistically significant changes occurring in terms of Pn, Gs, E or Ψ_S_ in comparison with the well-watered condition (80% SWC). Photosynthetic activity reached by all plants under well-watered conditions was lower than regular field activity due to the adaptation of leaves to moderate light [∼PPFD (Photosynthetically active Photon Flux Density) of 600 μmol of photons/(m^2^ × s)], with values between high and low light conditions obtained by Schubert et al. [48] under field conditions. 

### 3.2. The Effect of Water Stress on Grapevine Rootstock Genotypes

Under water deficit conditions (20% SWC), the *V. berlandieri × V. rupestris* hybrids (140Ru, 1103P and 110R) and the M-rootstocks and 41B rootstocks maintained high photosynthetic activity in comparison with the *V. riparia* × *V. berlandieri* hybrids and Schwarzmann rootstocks (Figure 1a). Besides photosynthesis, M-rootstocks and *V. berlandieri × V. rupestris* hybrids were more efficient in the use of water under limited conditions, showing higher iWUE values than *V. riparia* × *V. berlandieri* hybrids and 41B rootstocks (Figure 1b). On reducing the water availability, M3 and M4 rootstocks and most of the commercial rootstocks closed stomata, showing significant differences in Gs values compared to the well-watered condition. M4 and other rootstocks (110R, 161-49C, and SO4) significantly reduced both Gs and Pn values in response to water-stressed conditions (Figure 1). These genotypes are considered “plastic”, due to their ability to modify their performances under different environmental conditions [5,49,50]. However, M1 and 140Ru showed an “elastic” behavior, as they maintained unchanged Pn and iWUE levels under both well-watered and water-stressed conditions.

The genetic background of M-rootstocks and nine commercial grapevine rootstocks became discernible in their performances under water-deficit conditions (Figure 1 and Figure 2). In agreement with the literature [23,51,52], the *V. riparia* × *V. berlandieri* hybrids (161-49C, 420A, K5BB and SO4 rootstocks) showed lower tolerance to water stress than *V. berlandieri* × *V. rupestris* hybrids (1103P, 110R and 140Ru rootstocks), with lower Pn values. In Padgett-Johnson et al. [53], *V. rupestris* showed higher drought tolerance than *V. riparia* and *V. berlandieri.* Schwarzmann (*V. riparia* × *V. rupestris*) showed a performance similar to *V. riparia × V. berlandieri* hybrids, whereas 41B rootstock (*V. berlandieri* × *V. vinifera*), typically classified as a tolerant genotype, showed a behavior similar to *V. berlandieri* × *V. rupestris* hybrids. 

In our study, the performances of M-rootstocks (M1, M3 and M4), characterized by different genetic backgrounds, were similar to those shown by the *V. berlandieri* × *V. rupestris* hybrids 1103P and 110R. M4 (unknown × 1103 P) and 1103P rootstocks, both considered highly tolerant to water stress [23,54,55], showed similar performances (Figure 2). 

A recent study compared M4 to 1103P in grafting combination with Grechetto Gentile. The two combinations maintained similar water potential under water stress, though M4 showed higher photosynthesis and water use efficiency [56]. Galbignani et al. [57] found higher Pn values and higher instantaneous WUE values in Sangiovese grafted on M4 than grafted on SO4 under moderately water-stressed conditions. 

*Vitis* species possess the ability to show different strategic behaviors in response to drought [53]. In this study, three different strategies based on gas exchange and iWUE were identified in response to severe water-deficit conditions: (i) M-rootstocks (M1, M3 and M4) and 1103P and 110R rootstocks showed high Pn at limiting Gs values (Group A); (ii) Schwarzmann rootstock and *V. riparia × V. berlandieri* hybrids showed low Pn values at low Gs values (Group B); (iii) 140Ru and 41B rootstocks showed high Pn values without a reduction of Gs values (Group C) (Figure 2). 

The three groups reported differences in stem water potential under low SWC (Figure 3a), as well as in the expression of genes related to ABA biosynthesis and signaling (Figure 4).

### 3.3. Delineation of Group Strategies to Face Drought

Based on physiological performance under water-limiting conditions, the rootstocks were classified into three groups (A, B and C) (Figure 2). The same three clusters were clearly discriminated according to the expression of six genes related to the ABA pathway in both mild (50% SWC) and limiting (20% SWC) water-stressed conditions (Figure 4). ABA mediates many physiological responses of plants to drought, including avoidance as well as tolerance responses. It is synthesized in both roots and leaves [24]. In both organs, its levels increase upon exposure to drought and they are accompanied by major changes in gene expression and physiological responses, such as stomatal closure [17]. Differences among groups in physiological activity were only detected under water-limiting conditions (20% SWC), nevertheless, the three groups were clearly discriminated at mildly water stress (50% SWC) according to their gene expression (Figure 4). At 20% SWC, the discriminant function 1 correlated with *VvPP2C4* and *VvABF2* gene expression in leaves, involved in the ABA signal transduction [40,58,59]. Discriminant function 2 was mainly correlated with *VvZEP*, *VvNCED1* and *VvNCED2* gene expression in roots, involved in ABA biosynthesis [34,59].

Vines can use several strategies for drought adaptation, including avoidance, tolerance and resistance [60,61]. The expression of genes related to the ABA biosynthetic pathway helped to investigate the strategies adopted by groups to deal with the water deficiency. Group A rootstocks (M1, M3, M4, 1103P and 110R) experienced stress at 20% SWC (Figure 2), increasing the transcription of genes related to ABA biosynthesis, especially *VvNCED1* and *VvNCED2* (Figure 5b–c). However, they showed a low expression of genes linked to ABA signal transduction, showing negative values of discriminant function 1 (Figure 4b). The evidence that genes related to ABA signal transduction (*VvPP2C4*, *VvSnRK2.6*, *VvABF2*) showed low levels of gene expression at low Gs levels allows us to suppose that the stomatal closure in response to ABA increase might be associated with a different mechanism. An alternative way to achieve a fast increase in ABA content is via hydrolysis of the ABA-glucosyl ester (ABA-GE), an inactive glucose-conjugated form of ABA [59]. Nevertheless, Group A rootstocks reduced the stomatal conductance, despite which they retained high Pn activity, proving high water use efficiency (Figure 1b, Figure 2). Photosynthetic activity and stomatal closure involved reductions of both sub-stomatal CO_2_ concentration (Ci) and vapor pressure deficit (Vpd). This performance could be ensured by an efficient ROS detoxification system, for which gene expression was noticed for the M4 rootstock under water-stressed conditions by Corso et al. [48]. (Figure 3) The rootstocks clustered in Group A, including the M-rootstocks (M1, M3 and M4), adopted a *tolerant* strategy [61], preserving their physiological activity under water stress.

Rootstocks belonging to Group B (161-49C, 420A, K5BB, Schwarzmann and SO4) also reduced the physiological activity at 20% SWC (Figure 1b). Among genes related to ABA biosynthesis, they mainly increased transcripts of *VvAPF2, VvNCED2* and *VvPP2C4* genes (Figure 5c–d). According to the literature, the enhanced activity of *VvPP2C* genes during drought stress suggests that its primary role is in regulating ABA response [40,41,58]. As reported by Boneh et al. [40] and Rattanakon et al. [59], transcripts of *PP2C* genes increase to slow down the activation of the ABA signaling pathway that occurs from a rapid rise in the hormone itself. For Group B rootstocks, stomatal conductance decreased, as well as photosynthetic activity, showing low efficiency in water use (Figure 1b, Figure 2). For this group, low stomatal conductance seemed to buffer the drop in Ψ_S_ values at decreasing SWC levels (Figure 3a–b). (Figure 6) The strategy adopted by Group B genotypes under water-stressed conditions can be defined as *avoidance* [61], preserving Ψ_S_ by reducing the physiological activity through stomatal closure. The high water potential indicates that this could be adopted in long-term drought conditions.

Unlike other rootstocks, Group C rootstocks (140Ru and 41B) maintained the stomatal conductance under 20% SWC, allowing leaves to continue high photosynthetic activity (Figure 2), regardless of Ψ_S_ (Figure 3b). The physiological activity performed at 20% SWC could be related to the adaptation of genotype architecture to drought conditions, such as the vessel size [5,62,63]. (Figure 6) However, the expression of genes linked to ABA biosynthesis, especially *VvZEP*, rose at 50% SWC before decreasing at lower water availability (Figure 5a). Group C rootstocks showed a *resistant* strategy to water stress under water-limited conditions.

## 4. Material and Methods

### 4.1. Plant Material and Growth Conditions

The experiment was conducted in June 2017, under environmentally controlled conditions in a greenhouse at DiSAA (University of Milan). The greenhouse was equipped with supplementary light and a cooling system, with a 16 hr light [∼PPFD of 600 μmol of photons/(m^2^ × s)] and 8 hr dark photoperiod and a range of temperatures from 23 to 28 °C. A total of twelve grapevine rootstocks were analyzed: 1103P, 110R, 114Ru, 161-49C, 41B, 420A, K5BB, Schwarzmann, SO4, used worldwide, and the newly released M1, M3, M4. Nine biological repetitions per genotype were monitored. Two-year-old cuttings were used. The vines were grown in 4 L plastic pots, trained on 1 m stakes and placed in a randomized complete block design. The growth substrate was composed of 70% sand and 30% peat, supplemented with a layer of expanded clay aggregate on the bottom of the pot to avoid water flooding. During the phenological phase of budding, the plants were maintained in well-watered conditions in order to develop a well-expanded canopy. 

### 4.2. Irrigation Management and Treatments

Three treatments were performed: 80%, 50% and 20% soil water content (SWC). Per treatment, three plants were collected, which were considered as biological replications. The SWC was calculated using the gravimetric method, according to the formula suggested by Gardner et al. [64]:

(1) $$SWC=\frac{\left(\mathrm{fresh}\;\mathrm{weight}\;-\;\mathrm{dry}\;\mathrm{weight}\right)}{\mathrm{dry}\;\mathrm{weight}}\;100$$

where fresh weight refers to the soil weight at field capacity and dry weight to the soil dried in an oven at 105 °C for 48 h.

Each pot containing one plant was weighed daily for a period of 10 days. When SWC reached the values of 80%, 50% and 20%, plants were selected for measurement of physiological parameters and gene expression analysis. 

### 4.3. Plant Phenotyping

At each time point (80%, 50% and 20% SWC), gas exchange parameters and stem water potential (Ψ_S_) were evaluated in three different plants (replications) per rootstock. Both measurements were carried out between 11:00 a.m. and 2:00 p.m. solar time. 

Two fully expanded leaves (8th and 9th leaf) per plant were selected to measure gas exchange indicators: photosynthetic activity (Pn; μmol CO_2_ m^−2^ s^−1^), stomatal conductance (Gs; mmol H_2_O m^−2^ s^−1^) and transpiration (E; mmol H_2_O m^−2^ s^−1^). Gas exchange was measured with a leaf portable photosynthesis system (CIRAS-2, PP Systems, Amesbury, MA, USA) equipped with PLC6 (U) cuvette 18 mm circular (2.5 cm^2^ head plate), under constant saturating PPFD of 1500 µmol photons m^−2^ s^−1^, CO_2_ concentration of 300 μmol mol^−1^, block temperature of 25 °C and relative humidity between 60% and 70% allowing ~1.5 kPa of Vpd inside the leaf chamber. Intrinsic water use efficiency (iWUE) was calculated as the Pn/Gs ratio and expressed as μmol CO_2_ mol^−1^ H_2_O.

As suggested by Scholander et al. [65], Ψ_S_ (MPa) was calculated using the Scholander pressure chamber (Soil Moisture Equipment Corporation, Santa Barbara, CA, USA). The same leaves used to evaluate gas exchange were placed in a plastic bag wrapped in aluminum foil for 1 hr. Subsequently, they were excised with a razor blade and put in the Scholander chamber for the analysis. The Ψ_S_ value was recorded within 30 s from the cutting of the leaf by slowly pressurizing the chamber until sap came out from the cut end of the petiole. 

### 4.4. Gene Expression Analysis

After the in vivo measurements of physiological parameters at 80%, 50% and 20% SWC, the whole root system and fully expanded leaves (i.e., from the fifth to the eighth node of the primary shoot) of the same plants per rootstock were immediately sampled, frozen in liquid nitrogen and stored at −80 °C until RNA extraction. The total RNA was extracted from 100 mg of liquid nitrogen-ground tissue with a Spectrum™ Plant Total RNA (Sigma-Aldrich, Germany) commercial kit, according to the manufacturer’s instructions. To evaluate RNA quality, 260/230 and 260/280 ratios were checked via a NanoDrop Spectrophotometer (Thermo Scientific, MA, USA). For those samples showing a 260/230 absorbance ratio lower than 1.8, a lithium chloride treatment was carried out (as reported in De Lorenzis et al. [66]). RNA integrity was checked by electrophoresis on 1.5% agarose gel. RNA quantification was performed using a Qubit^®^ RNA HS Assay Kit by Qubit^®^ 3.0 Fluorometer (Life Technologies, Carlsbad, CA, USA). 

Total RNA (200 ng) was used to synthetize cDNA using 200 U of SuperScript^®^ III Reverse Transcriptase (Thermo Fisher) and 50 µM oligo(dT)_20_ primers in accordance with the manufacturer’s instructions. Six genes (Table 3) involved in the response to drought were evaluated via real-time RT-PCR. *VvZEP*, *VvNCED1* and *VVNCED2* genes were evaluated in roots and *VvPP2C4*, *VVSnRK2.6* and *VvABF2* genes were evaluated in leaves, based on previous evidence reporting that genes related to ABA biosynthesis are mainly associated with ABA increases in water-stressed roots [33,34], while genes related to the ABA signal transduction better discriminate the genotypes at leaf level [34]. Ubiquitin (UBI; [67]) and actin (ACT; [68]) were used as reference genes. RT-PCR was carried out in a 7300 Real-Time PCR System (Applied Biosystems, Foster City, CA, USA). For each reaction (20 µL), 200 nM of each primer, 2 µL of cDNA (1:100 dilution of the synthesis reaction), 1X SYBR Green Real-Time PCR Master Mix (Thermo Fisher) and water up to 20 µL were added. Thermal cycling involved pre-incubation at 95 °C for 3 min, followed by 40 cycles of 94 °C for 15 s, 58 °C for 20 s and 72 °C for 30 s. For detecting non-specific amplifications in cDNA samples, a melting cycle with temperatures ranging from 65 to 95 °C was performed. Each real-time RT-PCR reaction was completed in triplicate. After testing the suitability of ubiquitin and actin as reference genes, ubiquitin was selected to normalize the cycle threshold (Ct) values of all analyzed samples, due to a PCR efficiency value more similar to the ones observed for target genes (ranging from 93 to 97%). The expression of each gene in different genotypes and water conditions was calculated by comparing their 2^−ΔΔCt^ values [69].

### 4.5. Statistical Analysis 

Data were analyzed using Microsoft Office Excel and SPSS statistical software (IBM SPSS Statistics 24). A univariate ANOVA model was performed on phenotypical parameters (Pn, Gs, E and Ψ_S_) at *p* ≤ 0.05 after checking for the assumption of normality and homogeneity of variance. Post hoc comparisons were performed on phenotypical parameters (Pn, Gs, E and Ψ_S_) with Tukey’s post hoc test at *p* ≤ 0.05. Gene expression data were used to perform a discriminant analysis, using the values as independent variables and with equal prior probabilities. Groups were identified by a bi-plot of Pn and Gs using the available water-stressed threshold for Gs (50 mmol H_2_O m^−2^ s^−1^, based on Cifre et al. [49]) and the mean value for Pn. Differences among groups in terms of discriminant function scores and gene expression were detected by a univariate ANOVA model and Tukey’s post-hoc test at *p* ≤ 0.05. Correlation among phenotypical parameters and gene expression was determined by Pearson’s index at *p* = 0.05 (*) and *p* = 0.01 (**) and viewed as a heatmap.

## 5. Conclusions

In this study, the new M-rootstocks showed a reaction to water-stressed conditions similar to that of the 1103P and 110R rootstocks, two commercial genotypes typically classified as being highly tolerant. They adopted a tolerant strategy, increasing the transcripts of genes related to ABA biosynthesis, especially *VvNCED1* and *VvNCED2*, maintaining high water use efficiency under water-stressed conditions and preserving physiological activity even with low levels of stomatal conductance. Further studies will be necessary to confirm the performance of M-rootstocks under water stress in field conditions, evaluating rootstock/scion interactions. Nevertheless, a few new grapevine rootstock genotypes are not enough to face the challenges that modern viticulture will have to cope with in the future, therefore, new breeding programs have to be planned.

## Figures and Tables

**Table 1:** List of 12 grapevine rootstocks subjected to water limitation and information about their pedigree (based on Migliaro et al. [43]).

Rootstock	Pedigree
1103P	*V. berlandieri* cv. Resseguier nr. 2 × *V. rupestris* cv. Du Lot
110R	*V. berlandieri* cv. Boutin × *V. rupestris* cv. Du Lot
140Ru	*V. berlandieri* cv. Boutin × *V. rupestris* cv. Du Lot
161-49C	*V. berlandieri × V. riparia*
41B	*V. vinifera* cv. Chasselas × *V. berlandieri* cv. Planchon
420A	*V. berlandieri* × *V. riparia*
K5BB	*V. berlandieri* Resseguier nr. 2 × *V. riparia* cv. Gloire de Montpellier
M1	Kober 5BB × Teleki 5C (*V. berlandieri* cv. Planchon × *V. riparia*)
M3	Kober 5BB × Teleki 5C
M4	unknown × 1103 P
Schwarzmann	*V. riparia* × *V. rupestris*
SO4	*V. berlandieri* cv. Resseguier nr. 2 × *V. riparia* cv. Gloire de Montpellier

**Table 2:** Average value and standard deviation of physiological parameters for grapevine rootstock genotypes of Group A (1103P, 110R, M1, M3 and M4), Group B (161-49C, 420A, K5BB, Schwarzmann and SO4) and Group C (140Ru and 41B) at 20% soil water content (SWC). Groups are defined in Figure 2, according to the intrinsic water use efficiency. Pn = net photosynthesis (μmol CO_2_ m^−2^ s^−1^); Gs = stomatal conductance (mmol H_2_O m^−2^ s^−1^); E = transpiration (mmol H_2_O m^−2^ s^−1^); Ψ_S_ = stem water potential (MPa). Statistical differences among groups for each parameter are defined according to Tukey’s post hoc test at a *p*-value of 0.05.

Parameter	Group A				Group B				Group C			
Pn	2.93	±	0.66	a	1.47	±	1.15	b	3.68	±	1.62	a
Gs	35.80	±	13.40	a	28.26	±	16.16	a	67.25	±	36.23	b
E	0.77	±	0.26	a	0.66	±	0.36	a	1.38	±	0.67	b
Ψ_S_	−0.76	±	0.13	a	−0.59	±	0.14	b	−0.66	±	0.14	ab

**Table 3:** List of genes evaluated via real-time RT-PCR in roots and leaves of 12 own-rooted grapevine rootstocks grown under water deprivation.

Genes	Primer Sequence (5′ → 3′)	Reference	Tissue
*VvZEP*	F: GGTAAGAAGGAAAGGTTGC R: CAATAGGAGTCCCTGATTTGATGC	Hayes et al. [70]	roots
*VvNCED1*	F: TGCAGAGGACGAGAGTGTAA R: AGCTACACCAAAAGCTACGA	Hayes et al. [70]	
*VvNCED2*	F: ATGCTCAAACCGCCTCTGAT R: TCCCAAGCATTCCAGAGGTG	Lund et al. [71]	
*VvPP2C4*	F: TGGGCTTTGGGATGTTATGT R: TGTGCAGGAGTCTCATCAGC	Boneh et al. [40]	leaves
*VvSnRK2.6*	F: CACCAACCCACCTTGCTATT R: AAACGTGCCTCATCCTCACT	Boneh et al. [40]	
*VvABF2*	F: GGCACCCAGGCTAGTTAA R: GCAGAGTACACGCTAGATTG	Rossdeutsch et al. [34]	

## Supplementary Materials

The following are available online at https://www.mdpi.com/2223-7747/9/10/1385/s1, Figure S1. Effect of water availability decreasing (50 and 20% SWC; soil water content) in 12 own-rooted grapevine rootstocks on (a) net photosynthesis (Pn), (b) stomatal conductance (Gs), (c) transpiration (E) and (d) stem water potential (ΨS) in respect to the well-watered condition (80% SWC). ΨS values were normalized on well-watered condition values. Statistical differences among different water conditions for each parameter are defined according to Tukey post-hoc test at p-value 0.05. Well-watered plants were maintained at 80% SWC and compared to water-stressed plants at 50 and 20% SWC. At 50% SWC, Pn activity amounted to 3.90 μmol CO2 m-2 s-1, Gs to 124 mmol H2O m-2 s-1, E to 1.61 mmol H2O m-2 s-1 and ΨS to -0.42 MPa (about 113% in comparison to well-watered condition) (Figure 1). Average values of all physiological parameters were not significantly different in comparison with those of the well-watered condition. At 20% SWC, water availability significantly affected all the investigated physiological parameters (Figure 1). Plants reduced all the physiological parameters, showing significant differences in comparison to the well-watered plants: Pn was 2.47 μmol CO2 m-2 s-1 (about 37% less than 80% SWC); Gs was 38 mmol H2O m-2 s-1 (about 69% less than well-watered condition); E was 0.83 mmol H2O m-2 s-1 (about 48% less in comparison to well-watered condition); ΨS dropped to -0.67 MPa, Table S1: Average values and standard error of physiological parameters measured for 12 own-rooted grapevine rootstocks under both well-watered (80% soil water content, SWC) and water-stressed (20% SWC) conditions, Table S2: Average values and standard error of gene expression for six genes detected for 12 own-rooted grapevine rootstocks under both well-watered (80% soil water content, SWC) and water-stressed (20% SWC) conditions, Table S3: Standardized canonical discriminant function coefficients for function 1 and function 2 at 50 and 20% soil water content (SWC) for gene expression values of six genes detected in 12 own-rooted grapevine rootstocks.
