# Supplementary material for: How Do Novel M-Rootstock (Vitis Spp.) Genotypes Cope with Drought?
Source: Plants (Basel). 2020 Oct 17;9(10):1385. doi: 10.3390/plants9101385 (PMC7603061; doi:10.3390/plants9101385)
Supplement: Supplementary file 1 [file plants-09-01385-s001.pdf]

Table S1. Average values and standard error of physiological parameters measured for 12 own-rooted grapevine rootstocks under both well-watered (80% SWC; soil water content) and water-stressed (20% SWC) conditions.

Parameter	SWC [%]	1103P	110R	140Ru	161-49C	41B	420A	K5BB	M1	M3	M4	Schwarzmann	SO4
E	80	1.61 ± 0.24	2.56 ± 0.49	1.49 ± 0.19	1.74 ± 0.49	3.46 ± 0.25	0.98 ± 0.2	2.66 ± 0.41	1.78 ± 0.35	1.44 ± 0.17	1.71 ± 0.18	2.39 ± 0.37	2.41 ± 0.29
	50	1.35 ± 0.19	2.23 ± 0.33	1.1 ± 0.36	1.68 ± 0.47	2.45 ± 0.35	1.28 ± 0.28	1.35 ± 0.38	0.75 ± 0.12	2.78 ± 1.3	1.2 ± 0.55	0.93 ± 0.09	2.2 ± 0.64
	20	0.55 ± 0.05	0.78 ± 0.06	1.38 ± 0.19	0.38 ± 0.09	1.38 ± 0.48	1.03 ± 0.23	0.67 ± 0.15	0.85 ± 0.14	0.78 ± 0.19	0.88 ± 0.15	0.48 ± 0.14	0.78 ± 0.14
Gs	80	104 ± 25	84 ± 7	65 ± 6	83 ± 31	159 ± 19	52 ± 7	70 ± 14	53 ± 10	93 ± 12	85 ± 8	164 ± 70	122 ± 20
	50	116 ± 15	192 ± 48	75 ± 28	108 ± 38	173 ± 37	75 ± 18	113 ± 40	45 ± 9	275 ± 140	91 ± 40	57 ± 6	165 ± 47
	20	30 ± 3	31 ± 2	60 ± 8	15 ± 3	75 ± 26	41 ± 10	24 ± 5	41 ± 6	42 ± 12	36 ± 7	24 ± 9	37 ± 7
Pn	80	5.23 ± 1.13	5.1 ± 0.15	4.13 ± 1.15	3.53 ± 0.89	5.63 ± 0.34	3.33 ± 0.37	2.23 ± 0.3	3.03 ± 0.62	4.03 ± 0.41	4.23 ± 0.18	3.6 ± 0.75	5.6 ± 0.61
	50	4.28 ± 0.64	6.68 ± 1.64	3.8 ± 0.69	4.03 ± 1.08	5.65 ± 1.27	2.55 ± 1.28	4.65 ± 0.51	2.5 ± 0.58	2.63 ± 0.45	1.63 ± 0.83	3.9 ± 0.51	4.48 ± 1.1
	20	2.7 ± 0.4	2.93 ± 0.19	4.28 ± 0.62	0.6 ± 0.32	3.08 ± 0.95	1.25 ± 0.51	1.2 ± 0.67	3.13 ± 0.26	3.23 ± 0.5	2.65 ± 0.29	2.13 ± 0.91	2.13 ± 0.08
iWUE	80	50.8 ± 3.45	61.55 ± 5.57	67.23 ± 22.77	56.65 ± 19.73	36.53 ± 5.26	71.47 ± 11.61	37.79 ± 14.44	65.48 ± 22.86	44.43 ± 6.63	50.92 ± 6.94	35.83 ± 17.41	47.22 ± 6.08
	50	37 ± 2.85	37.51 ± 10.76	70.74 ± 18.62	46.25 ± 14.52	37.77 ± 11.13	38.2 ± 14.17	66.24 ± 23.96	57.93 ± 8.86	35.18 ± 20.89	33.53 ± 14.86	71.02 ± 10.43	36.15 ± 10.98
	20	90.43 ± 7.9	94.79 ± 5.21	73.14 ± 9.97	32.26 ± 15.91	59.7 ± 20.23	41.12 ± 16.33	54.91 ± 28.12	84.03 ± 18.38	89.85 ± 16.15	79.05 ± 11.58	70.84 ± 26.69	64.04 ± 12.66
Ψ_S	80	0,27 ± 0,03	0,38 ± 0,1	0,27 ± 0,03	0,39 ± 0,12	0,41 ± 0,02	0,42 ± 0,11	0,4 ± 0,18	0,36 ± 0,13	0,46 ± 0,12	0,31 ± 0,07	0,45 ± 0,1	0,36 ± 0,11
	50	0,46 ± 0,14	0,44 ± 0,04	0,36 ± 0,04	0,36 ± 0,04	0,57 ± 0	0,32 ± 0	0,51 ± 0,05	0,36 ± 0,04	0,44 ± 0,04	0,44 ± 0,12	0,4 ± 0	0,32 ± 0
	20	0,92 ± 0,13	0,86 ± 0,07	0,53 ± 0	0,46 ± 0,07	0,79 ± 0	0,46 ± 0,07	0,79 ± 0	0,73 ± 0,07	0,66 ± 0	0,66 ± 0	0,66 ± 0	0,59 ± 0,07

Table S2. Average values and standard error of gene expression for six genes detected for 12 own-rooted grapevine rootstocks under both well-watered (80% SWC; soil water content) and water-stressed (20% SWC) conditions.

Gene	SWC [%]	1103P	110R	140Ru	161-49C	41B	420A	K5BB	M1	M3	M4	Schwarzmann	SO4
VvABF2	80	0.95 ± 0.02	1.01 ± 0.02	1.01 ± 0	1 ± 0.09	0.99 ± 0.05	1.05 ± 0.04	0.99 ± 0.03	1.12 ± 0.04	1.04 ± 0.04	0.97 ± 0.12	1 ± 0.03	0.97 ± 0.12
	50	1.16 ± 0.14	2.03 ± 0.41	2.66 ± 0.2	0.89 ± 0.02	1.57 ± 0.03	1.34 ± 0.12	1.59 ± 0.12	1.21 ± 0.08	0.75 ± 0.04	1.45 ± 0.26	2.05 ± 0.3	1.16 ± 0.09
	20	0.96 ± 0.05	1.24 ± 0.17	1.89 ± 0.13	8.86 ± 0.07	1.76 ± 0.04	1.07 ± 0.08	1.13 ± 0.2	0.32 ± 0.02	3.18 ± 0.47	2.26 ± 0.31	2.97 ± 0.13	0.87 ± 0.05
VvNCED 1	80	0.99 ± 0.15	1.47 ± 0.07	1.01 ± 0.06	0.98 ± 0.12	1.19 ± 0.09	0.94 ± 0.08	1.07 ± 0	1.09 ± 0.04	0.92 ± 0.15	0.98 ± 0.05	0.98 ± 0.11	1.02 ± 0.02
	50	1.22 ± 0.02	33.15 ± 5.09	1.71 ± 0.13	3.86 ± 0.42	13.42 ± 1.57	1.93 ± 0.01	10.92 ± 3.19	37.54 ± 9.66	4.44 ± 0.74	4.99 ± 0.15	0.87 ± 0.04	2.94 ± 0.72
	20	2.5 ± 0.57	21.15 ± 5.1	1.31 ± 0.09	9.5 ± 1.66	2.12 ± 0.4	3.69 ± 0.61	8.61 ± 1.33	64.74 ± 8.19	5.5 ± 0.02	4.34 ± 0.65	4.62 ± 0.49	1.93 ± 0.41
VvNCED 2	80	1 ± 0.14	1.11 ± 0.15	1.03 ± 0.16	1.2 ± 0.29	0.98 ± 0.06	0.98 ± 0.1	0.96 ± 0.08	0.95 ± 0.05	0.92 ± 0.06	1.08 ± 0.03	1.08 ± 0.01	0.89 ± 0.12
	50	0.96 ± 0.01	0.87 ± 0.15	1.13 ± 0.22	16.83 ± 2.13	14.3 ± 2.49	1.45 ± 0.08	15.93 ± 1.78	63.72 ± 12.09	2.77 ± 0.29	2.76 ± 0.11	2.81 ± 0.17	29.11 ± 5.36
	20	2.95 ± 0.21	1.54 ± 0.15	1.27 ± 0.01	29.5 ± 7.4	2.82 ± 0.67	5.79 ± 1.02	13.3 ± 1.95	35.18 ± 6.21	4.25 ± 0.46	4.86 ± 0.76	4.21 ± 0.07	43.94 ± 9.98
VvPP2C4	80	0.94 ± 0.07	1.03 ± 0.04	1 ± 0	1.03 ± 0.12	0.93 ± 0.06	1.04 ± 0.03	1.06 ± 0.05	1.06 ± 0.04	1.03 ± 0.04	0.94 ± 0.08	1.33 ± 0.29	1.08 ± 0.02
	50	1.36 ± 0.15	3.65 ± 0.47	3.14 ± 0.1	1.38 ± 0.28	2.02 ± 0.2	11.72 ± 2.05	1.11 ± 0.09	0.82 ± 0.02	1.05 ± 0.02	0.97 ± 0.01	1.43 ± 0.06	1.68 ± 0.05
	20	2.9 ± 0.39	2.07 ± 0.28	3.59 ± 0.41	8.39 ± 0.13	5.65 ± 0.09	32.46 ± 2.02	2.36 ± 0.08	0.98 ± 0.11	1.15 ± 0.04	4.62 ± 1.01	6.45 ± 0.32	5.11 ± 0.89
VvSnRK2 6	80	0.94 ± 0.03	1.01 ± 0.05	1 ± 0	1.01 ± 0.07	1.01 ± 0.05	1.02 ± 0.04	1 ± 0.02	1.1 ± 0.07	0.91 ± 0.07	1.04 ± 0.04	1.01 ± 0.11	0.96 ± 0.11
	50	1.02 ± 0.1	1.11 ± 0.17	1.36 ± 0.01	0.82 ± 0.09	0.93 ± 0.04	1.76 ± 0.25	1.27 ± 0.16	1.47 ± 0.19	1.17 ± 0.1	0.87 ± 0.06	1.97 ± 0.27	1.4 ± 0.14
	20	1.17 ± 0.06	1.57 ± 0.16	1.3 ± 0.05	0.7 ± 0.02	1.32 ± 0.04	2.56 ± 0.69	0.95 ± 0.03	1.29 ± 0.04	1.77 ± 0.39	1.86 ± 0.19	3.26 ± 0.25	0.98 ± 0.08
VvZEP	80	0.91 ± 0.07	0.86 ± 0.06	0.99 ± 0.1	1.05 ± 0.01	0.94 ± 0.1	1 ± 0.11	1.05 ± 0.07	1.06 ± 0	1 ± 0.02	1.1 ± 0.1	0.98 ± 0.06	1.22 ± 0.03
	50	1.36 ± 0.09	1.1 ± 0.06	1.44 ± 0.04	2.39 ± 0.46	31.13 ± 8.97	1.29 ± 0.11	8.38 ± 1.71	11.8 ± 0.56	2.69 ± 0.26	1.3 ± 0.36	1.31 ± 0.11	2.07 ± 0.4
	20	0.86 ± 0.06	1.3 ± 0.06	1.19 ± 0.01	2.18 ± 0.34	0.72 ± 0.06	1.82 ± 0.25	6.79 ± 1.25	10.4 ± 0.14	4.6 ± 0.6	1.2 ± 0.16	1.39 ± 0.17	1.24 ± 0.31

Table S3. Standardized canonical discriminant function coefficients for Function 1 and Function 2 at 50 and 20% SWC (soil water content) for gene expression values of six genes detected in 12 own-rooted grapevine rootstocks.

	**50% SWC**		**20%SWC**	
	**Function 1**	**Function 2**	**Function 1**	**Function 2**
VvABF2	1.402	-0.137	0.374	0.13
VvNCED1	-1.174	-1.04	-1.298	0.356
VvNCED2	0.733	0.767	1.203	0.346
VvPP2C4	0.462	0.373	0.625	-0.098
VvSnRK26	-0.828	0.354	0.592	0.548
VvZEP	0.924	-0.207	0.686	0.531

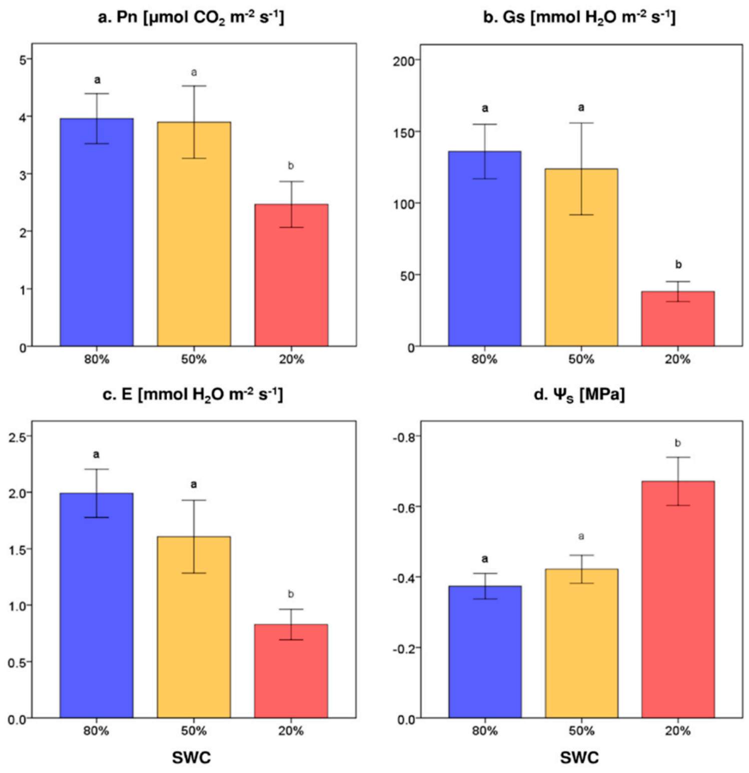

Figure S1. Effect of water availability decreasing (50 and 20% SWC; soil water content) in 12 own-rooted grapevine rootstocks on (a) net photosynthesis (Pn), (b) stomatal conductance (Gs), (c) transpiration (E) and (d) stem water potential (Ψ_S) in respect to the well-watered condition (80% SWC). Ψ_S values were normalized on well-watered condition values. Statistical differences among different water conditions for each parameter are defined according to Tukey post-hoc test at *p*-value 0.05. Well-watered plants were maintained at 80% SWC and compared to water-stressed plants at 50 and 20% SWC. At 50% SWC, Pn activity amounted to 3.90 μmol CO_2 m^{-2} s^{-1}, Gs to 124 mmol H_2O m^{-2} s^{-1}, E to 1.61 mmol H_2O m^{-2} s^{-1} and Ψ_S to -0.42 MPa (about 113% in comparison to well-watered condition) (Figure 1). Average values of all physiological parameters were not significantly different in comparison with those of the well-watered condition. At 20% SWC, water availability significantly affected all the investigated physiological parameters (Figure 1). Plants reduced all the physiological parameters, showing significant differences in comparison to the well-watered plants: Pn was 2.47 μmol CO_2 m^{-2} s^{-1} (about 37% less than 80% SWC); Gs was 38 mmol H_2O m^{-2} s^{-1} (about 69% less than well-watered condition); E was 0.83 mmol H_2O m^{-2} s^{-1} (about 48% less in comparison to well-watered condition); Ψ_S dropped to -0.67 MPa.
